# Exploring the decoy effect to guide tobacco treatment choice: a randomized experiment

**DOI:** 10.1186/s13104-019-4873-0

**Published:** 2020-01-02

**Authors:** Erin S. Rogers, Elizabeth A. Vargas, Elizabeth Voigt

**Affiliations:** 1 Department of Population Health, New York University School of Medicine, 180 Madison Ave, New York, NY 10016 USA; 2 VA NY Harbor Healthcare System, 423 East 23rd Street, New York, NY 10010 USA

**Keywords:** Smoking, Smoking cessation, Counseling

## Abstract

**Objectives:**

Guidelines recommend that smokers participate in four or more counseling sessions when trying to quit, but smokers rarely engage in multiple sessions. The “decoy effect” is a cognitive bias that can cause consumer preferences for a “target” product to change when presented with a similar but inferior product (a “decoy”). This study tested the use of a decoy to guide smokers’ selection of a target number of counseling sessions. During an online survey, adult tobacco users (N = 93) were randomized to one of two groups that determined the answer choices they saw in response to a question assessing their interest in multi-session cessation counseling. Group A choose between two sessions or a “target” of five sessions. Group B was given a third “decoy” option of seven sessions. Binary logistic regression was used to compare groups on the proportion of participants selecting the “target.”

**Results:**

Among 90 participants with complete data, a decoy effect was not found. There was no significant difference between groups in the proportion of participants selecting the target of five sessions (47% in Group B vs. 53% in Group A; aOR = 0.76, 95%CI 0.48–1.19).

*Trial Registration* This study was retrospectively registered at clinicaltrials.gov on December 13, 2019 (NCT04200157)

## Introduction

Smoking is the leading preventable cause of mortality in the U.S [1]. Effective smoking cessation treatments exist, including multi-session telephone counseling [2]. The United States Public Health Services (USPHS) guidelines recommend that smokers receive at least four sessions of counseling for optimal impact [2]. Unfortunately, most smokers do not use any counseling when trying to quit and even fewer participate in the recommended number of sessions [3–5].

The fields of marketing and behavioral economics may provide strategies for increasing the proportion of smokers who choose to participate in the recommended number of counseling sessions. Research shows that consumers evaluate the value of competing product options dependently and dimensionally—weighing multiple factors at a time, such as quality versus costs—and the context in which products are offered impacts consumer choice [6]. For example, adding a third product option that is similar (but inferior) to a “target” option enhances consumer preference for the “target” relative to a “competitor” product. This phenomenon is called “the decoy effect” (or the “the asymmetrical dominance effect”) and has been widely studied as a method for guiding consumers toward “target” products in hypothetical contexts, including brand marketing [7, 8], alcohol purchases [9] and consumer travel [10].

Only one study to our knowledge has tested the decoy effect as a means to guide people toward healthy treatment offerings. In two online experiments, Stoffel et al. [11] found that the inclusion of a decoy hospital option increased the probability of people choosing to receive colorectal cancer screening at a target hospital. Their study provided proof of concept that decoys can positively impact patient choice and potentially downstream health outcomes. The purpose of this study was to conduct the first test of whether a decoy effect can guide smokers to select a target number of counseling sessions.

## Main text

### Methods

#### Design and participants

The study used a randomized controlled study design. People aged 18 years or older who had smoked a cigarette in the prior 7 days were eligible for the study. ResearchMatch was used to recruit participants. ResearchMatch is a national web-based registry of people who have expressed interest in participating in research studies [12]. ResearchMatch’s volunteer database was queried for people ≥ 18 years old who self-reported current tobacco use. ResearchMatch sent an email to potential participants with a description of the study and a link to click if they agree to be contacted by the study. People who agreed to be contacted were sent a link to complete an online survey through a secure REDCap system. Non-respondents were sent up to two email reminders to complete the survey.

#### Procedures

The survey included a consent cover letter and a question confirming that the participant had smoked at least once cigarette (“even a puff”) in the prior 7 days. The survey lasted 5–10 min. Participants were emailed a $5 Amazon.com gift card for completing the survey.

#### Study groups

**Fig. 1:**
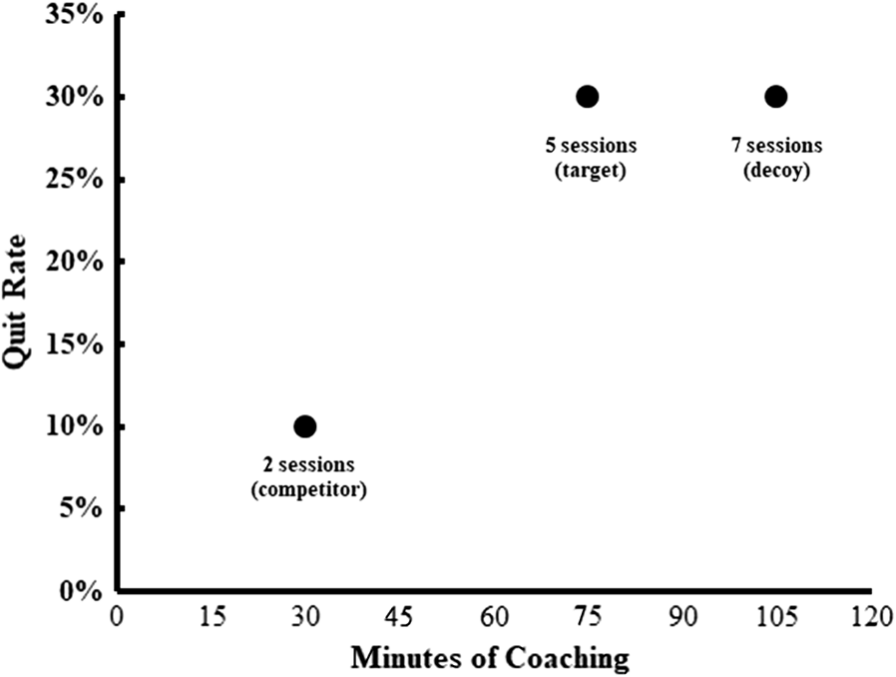
Operationalization of the decoy: time cost (minutes of counseling) and estimated quit rates of the counseling options presented to participants

Using a group allocation table uploaded into REDCap’s randomization module, participants were randomized 1:1 into one of two groups that determined the answer choices they saw in response to a question assessing their interest in tobacco cessation counseling sessions. Time costs have been previously used effectively in research on the decoy effect, and in smoking cessation interventions, abstinence rates can be conceptualized as the final “product” or benefit to the consumer. Therefore, to create our competitor, target, and decoy response options, we varied the time costs (minutes of counseling) and estimated abstinence rates of each option. Figure 1 displays the response options along these two dimensions. Two counseling sessions was selected to serve as the competitor choice in this experiment to coincide with a common number of sessions completed during real-world cessation studies [3–5]. We created a target of five sessions to align with the USPHS guidelines [2]. To create a decoy option, we decided to increase the target’s time cost by two sessions, while keeping the abstinence rates in the two groups the same. Therefore, the decoy was asymmetrically dominated by the target in that it would require additional time with no expected increases in quit rates. We made the decision to increase the decoy by two sessions instead of one session, in order to make the time cost difference (i.e., the inferiority) of the decoy salient to participants. In accordance with asymmetrical dominance theory, we decided to increase the decoy by only two sessions instead of three or more sessions, so that the decoy would be more similar to the target than the competitor (which was three sessions less than the target).

Participants randomized to Group A chose between two sessions or five sessions. Participants randomized to Group B chose between two, five, or seven sessions. The survey displayed the time cost and estimated abstinence rates next to the response options to make the dominance of the decoy salient to Group B. Participants were not told that we were testing the decoy effect.

#### Additional survey measures

The survey also asked participants sociodemographic questions (age, gender, race, ethnicity, education, marital status, household income), number of cigarettes smoked per day, motivation to quit (using a 1–10 scale), and whether participants had ever used telephone cessation counseling.

#### Outcomes and sample size

Our primary outcome was the percent of participants who selected the target of five sessions. We hypothesized that participants in Group B would be more likely to select the target of five sessions than participants in Group A. We aimed to enroll 100 participants, with 50 allocated to each group. If 50% of participants in Group A selected the target, a sample of 100 participants would give us 80% power at α = 0.05 to detect a significant group difference if 78% or more of Group B participants selected the target.

#### Analysis

Participants with complete survey data were included in the final analysis. Analyses were conducted with SPSS version 23. Descriptive statistics (means, standard deviations, and frequencies) were run to characterize the sample on sociodemographic and tobacco variables. We used t-tests and Chi square tests to compare the two groups on sociodemographic and tobacco variables. We then calculated the proportion of participants in each group that selected the competitor, target, or decoy response options. To estimate the decoy effect, we used binary logistic regression to compare the proportion of participants selecting the target option when the decoy was present (Group B) versus when the decoy was not present (Group A). We ran an unadjusted regression model and then an adjusted regression model controlling for participant characteristics that differed between groups at *p *< .05.

### Results

Of the 156 people who agreed to receive a study invitation through ResearchMatch, 93 (59.6%) completed the survey’s consent page and were eligible (i.e., were current smokers). Participants were randomized to Group A (n = 47) or Group B (n = 46). Forty-five participants in each group completed the survey and were included in the final analysis.

Table 1 displays participant characteristics. Participants were on average 40.4 (SD = 13.8) years old and were mostly female and non-Hispanic White. The sample was diverse with respect to education and marital status. Thirty-five percent had a high school education or less, 42% had an Associate’s degree or some college completed, and 33% had a Bachelor’s degree or higher. Thirty-six percent of participants were married, 32% had never been married, and 31% were divorced, separated or widowed. Participants had an average annual income of $59,286 (SD = 58,078). Participants smoked on average 10.9 (SD = 7.2) cigarettes per day. On a scale of 1–10, participants had an average level of motivation to quit of 5.7 (SD = 2.5), and 13% had previously tried a smoking cessation quitline. Participants in Group B were significantly more motivated to quit than participants in Group A (6.3 vs. 5.1, on a scale of 0–10, *p *< .05), so we controlled our analysis by motivation.

**Table 1:** Participant characteristics

Variable	Total (N = 90)	Group A (n = 45)	Group B (n = 45)	*P* value±
Age	40.4 (13.8)	40.9 (13.1)	39.8 (14.5)	0.68
Sex				0.08
Male	26 (29%)	9 (20%)	17 (38%)	
Female	62 (69%)	34 (76%)	28 (62%)	
Other	2 (2%)	2 (4%)	0 (0%)	
Race				0.62
White	67 (74%)	35 (78%)	32 (71%)	
Black	13 (14%)	6 (13%)	7 (16%)	
Other	10 (24%)	4 (9%)	6 (13%)	
Hispanic ethnicity	7 (8%)	3 (7%)	4 (9%)	1.00
Education				0.23
Some high school	1 (1%)	1 (2%)	0 (0%)	
High school/GED	21 (34%)	9 (20%)	12 (27%)	
Associate’s degree/some college	38 (42%)	16 (36%)	22 (49%)	
Bachelor’s degree	20 (22%)	14 (31%)	6 (13%)	
Graduate degree	10 (11%)	5 (11%)	5 (11%)	
Marital status				0.42
Married/living with partner	32 (36%)	16 (36%)	16 (16%)	
Divorced/widowed/separated	28 (31%)	16 (36%)	12 (27%)	
Never married	29 (32%)	12 (27%)	17 (38%)	
Annual income	$59, 286.3 (58, 078.1)	$48, 620.9 (45, 720.4)	$69, 697.7 (66, 947.5)	0.10
Cigarettes per day	10.9 (7.2)	11.6 (7.4)	10.1 (7.0)	0.33
Motivation to quit	5.7 (2.5)	5.1 (2.4)	6.3 (2.5)	0.03
Tried quitline counseling before	12 (13%)	4 (9%)	8 (18%)	0.35
Number of quitline calls	1.6 (1.1)	1.7 (1.2)	1.6 (1.2)	0.96

± Groups were compared using Chi square for categorical variables and t-tests for continuous variables

**Fig. 2:**
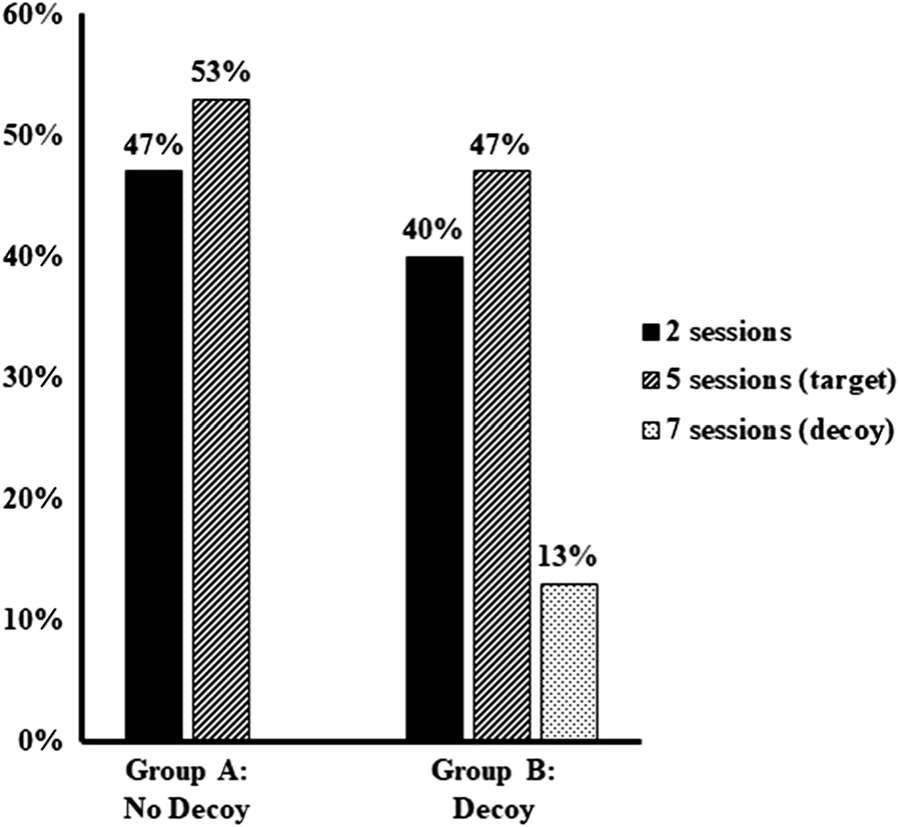
The proportion of participants selecting the counseling options with the decoy (Group B) and without the decoy (Group A)

Figure 2 displays the percent of people in each group that selected the response options. There was no significant difference between groups in the proportion of participants selecting the target of five sessions (47% in Group B versus 53% in Group A) in the unadjusted model (OR = 0.88, 95%CI 0.58–1.32) or when adjusting for group differences in motivation to quit (aOR = 0.76, 95%CI 0.48–1.19).

### Discussion

In this hypothetical choice exercise, the hypothesis that introducing a decoy option would enhance participant selection of a target number of counseling sessions was not supported. Participants randomized to view a decoy number of sessions selected the target at a similar rate as participants who were not given the decoy option. These results are inconsistent with asymmetrical dominance theory and prior research finding a significant impact of introducting decoys on consumer choice [8, 10].

The current study was only the second test to our knowledge of the decoy effect during the selection of a health behavior that is generally in low demand (i.e., behavioral cessation counseling). It is possible that undesired behaviors may be more resistent to the cognitive impact of a decoy than desirable purchases, such as vacation packages or alcohol [9, 10]. In Stoffel et al.’s study [11] of the impact of decoys on hypothetical colorectal cancer screening preferences, they found that the decoy was most impactful on preferences when it was strongly dominated by the target in both experimental dimensions (time cost and benefit) [11]. In the current study, the decoy was dominated by the target in only one dimension (time cost). Therefore, the decoy may not have been strong enough or participants may not have perceived the domination of the decoy as intended. Participants may have also viewed the “minutes of counseling” not as a time cost, but as a benefit. Therefore, the optimal cost/benefit ratio of the target option may not have been perceived by participants as intended. Additional research may be needed to explore other methods for modifying smokers’ perceptions of decoy treatment options in laboratory and real-world settings.

This was also the first test to our knowledge of the potential of decoys to influence consumer engagement in a behavior that often evolves over time (counseling participation). All prior research on this phenomenon tested decoys during the selection of discrete alternatives (e.g., one hospital over another, one vacation package over another). It is possible that decoys are only effective at guiding discrete choices. Future research may explore the decoy effect in the context of discrete tobacco treatment choices, such as nicotine replacement therapy purchases or initial sign-up for a text-messaging cessation program.

### Conclusions

Introducing a decoy option did not impact smokers’ selection of tobacco counseling options during an online survey. Future research may be needed to test the phenomenon in real-world settings and with other types of tobacco treatment options (e.g., discrete engagement).

## Limitations

This study is limited by its relatively small sample of mostly White, female smokers recruited through an online registry of people interested in participating in research. The study also tested a hypothetical choice of just one type of smoking cessation treatment. Results may not extend to other populations or how smokers may engage in a real-world environment.

## Data Availability

The individual de-identified data can be requested from the corresponding author.

## Abbreviations

SD: standard deviation
USPHS: United States Public Health Services
